# Mechanical and optical properties of ultralarge flakes of a metal–organic framework with molecular thickness†
†Electronic supplementary information (ESI) available: Extended experimental details, additional Figures and theoretical calculations. See DOI: 10.1039/c4sc03115f



**DOI:** 10.1039/c4sc03115f

**Published:** 2015-02-16

**Authors:** Cristina Hermosa, Benjamin R. Horrocks, José I. Martínez, Fabiola Liscio, Julio Gómez-Herrero, Félix Zamora

**Affiliations:** a Departamento de Química Inorgánica , Universidad Autónoma de Madrid , 28049 Madrid , Spain . Email: felix.zamora@uam.es; b Departamento de Física de la Materia Condensada , Universidad Autónoma de Madrid , 28049 Madrid , Spain . Email: julio.gomez@uam.es; c Chemical Nanoscience Laboratory , School of Chemistry , Newcastle University , UK; d Instituto de Ciencia de Materiales CSIC , 28049 Madrid , Spain; e CNR-IMM , Instituto per la Microelettronica e Microsistemi , via P. Gobetti 101 , I-40129 Bologna , Italy; f Condensed Matter Physics Center (IFIMAC) , Universidad Autónoma de Madrid , 28049 Madrid , Spain

## Abstract

The isolation of 2D-materials is already a success for graphene, graphene oxide, boron nitride and a few clays or metal chalcogenides, however despite the fact that some of them show very interesting physical properties, they lack useful functionalities. Metal–Organic Frameworks (MOFs) are multifunctional materials showing a wide range of physical and chemical properties that can be structurally designed by suitable selection of their building-blocks. This strategy may allow the production of layers with a variety of useful electronic and molecular recognition functionalities. Herein we isolate 2D-MOF flakes with areas of hundreds of square microns and an excellent control of the molecular thickness (from single up to *ca.* 50 layers). The samples exhibit such good photoluminescence and mechanical properties as to allow free-standing characterization of few layers’ flakes.

## Abstract 2

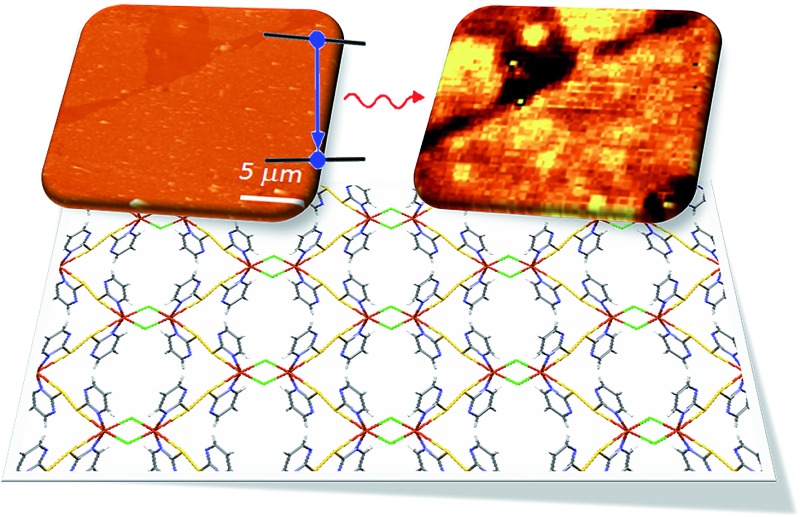
The red emission on isolated 2d-mof flakes with areas of square microns and molecular thicknesses (from single up to *ca.* 50 layers) has been characterized. Free-standing flakes have also been produced and their mechanical and optical properties studied.

## Introduction

Two-dimensional (2D) materials have attracted increasing attention in the last few years due to their potential expected applications.^1^ Graphene, a single layer of graphite, represents the first material of this kind.^2^ The fascinating physical properties^3^ and potential applications of graphene^4,5^ have stimulated the development of a number of 2D related materials including graphene oxide,^6,7^ BN,^8^ MoS_2_,^9^ and clays.^10^ However all of them show a rather limited or no chemical design and functionalities. Recently, it has been pointed out that covalent polymers,^11^ and layered covalent organic frameworks (COFs)^1,12,13^ or metal–organic frameworks (MOFs)^14^ could bring *à la carte* 2D materials with a variety of architectures, pre-designed cavities, and chemical functionalities.^15^ Even more importantly, suitable selection of the initial building blocks should enable preparation of multifunctional materials with interesting physical and chemical properties. In particular, MOFs are crystalline porous materials formed by linking organic molecules with metal fragments. They show a large number of properties and potential applications ranging from gas storage and separation, molecular sieves and catalysis to sensing.^16,17^ Additionally they have been used as a source of nanomaterials for the production, for instance, of nanoparticles^18^ and nanowires.^19^ The expected potential of metal–organic sheets to provide novel 2D-materials has triggered global research interest.^1,13^ Indeed, intensive attempts to generate 2D-layers of MOFs have been reported, applying different interesting strategies to this purpose. Thus, bottom-up procedures based on on-surface synthesis have been used to produce layers of MOFs but, unfortunately, the lateral dimensions of these structures are, so far, too small to expect relevant sheet-like properties;^20,21^ furthermore, they cannot yet be isolated and manipulated. Additionally, layer formation at the air–water interphase and subsequent deposition on surfaces has been proved as an alternative for production of MOFs exceeding micron lateral dimensions.^22–24^ However, although free-standing layers on TEM grids have demonstrated neither mechanical nor any other physical properties, they have been reported.

Alternatively, the top-down approach based on liquid phase exfoliation (LPE) of crystals of layered MOFs assisted by ultrasound has been developed as an alternative procedure.^25^ Some examples following this top-down approach have been recently published, but in all of them the lateral dimensions of the layers have precluded both free-standing isolation and physical characterization.^26–28^

Along this line we have designed a new laminar MOF of formula [Cu(μ-pym_2_S_2_)(μ-Cl)]_*n*_ (pymS_2_ = dipyrimidindisulfide), showing an interlayer interaction so weak that it can be delaminated just by interaction with an excess of solvent.^29^ Despite the fact that the bulk material showed intense red emission, the physical characterization of the layers of [Cu(μ-pym_2_S_2_)(μ-Cl)]_*n*_ was not possible due to their reduced lateral size. In this work, we have been able to set up a method to produce ultra-large layers with control over the thickness that has allowed us to obtain free-standing few-layers flakes of this MOF and characterize their mechanical properties and its emission in 2D-materials ranging from single to a few layers. The results show the high potential of several chemically designed layered materials, such as MOFs, to produce novel 2D layered functional polymers.

## Results and discussion

### Flakes production and characterization

Prismatic crystals [Cu(μ-pym_2_S_2_)(μ-Cl)]_*n*_·*n*MeOH (pymS_2_ = dipyrimidindisulfide) were synthesized by slow evaporation of a dipyrimidinedisulfide MeOH : MeCN solution into a methanolic solution of CuCl_2_·2H_2_O (ESI† for details).

**Fig. 1:**
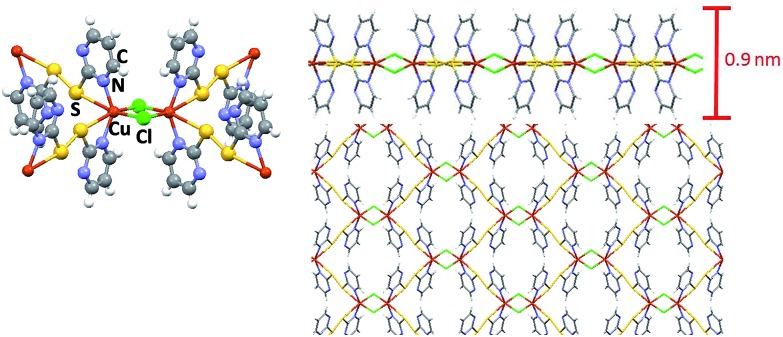
Representation of the basic components and structure of a layer of [Cu(μ-pym_2_S_2_)(μ-Cl)]_*n*_·*n*MeOH obtained by X-ray diffraction.

**Fig. 2:**
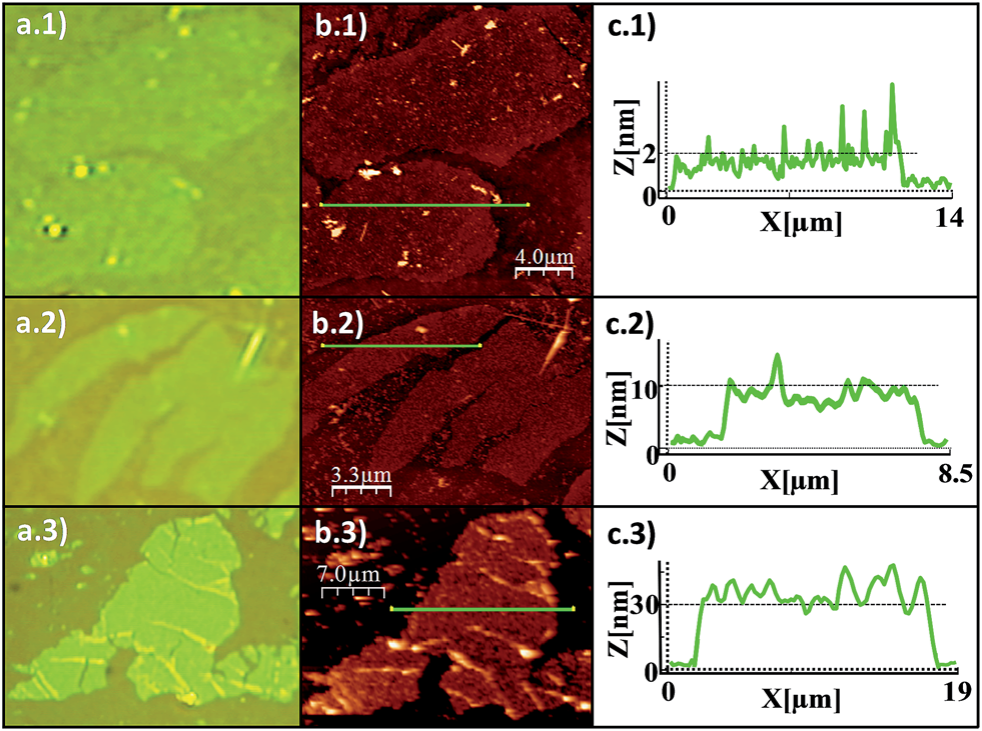
Morphological characterization of layers of [Cu(μ-pym_2_S_2_)(μ-Cl)]_*n*_·*n*MeOH on SiO_2_. Optical (a1–a3) and AFM topographic images (b1–b3) with their height profiles (c1–c3) showing thickness ranging from: single layer (2 nm, top), 14 layers (10 nm, middle) and 48 layers (30 nm, bottom).

Fig. 1 shows schematic views of the structure of the MOF [Cu(μ-pym_2_S_2_)(μ-Cl)]_*n*_·*n*MeOH in which the presence of a layered structure with cavities containing solvent molecules is remarkable. Large orange crystals of [Cu(μ-pym_2_S_2_)(μ-Cl)]_*n*_·*n*MeOH were suspended in water, then sonicated and centrifuged to obtain a homogeneous suspension of larger surface area flakes. The integrity of the material obtained after sonication was confirmed by X-ray diffraction analysis, Fig. S5.† The productions of these large lateral dimensions are a consequence of the size of the starting crystals (dimensions *ca.* 125–60 × 45–80 × 35–70 μm^3^) which are significantly bigger than those previously used.^29^ These improvements in the dimensions of the laminar crystals combined with control of the exfoliation and centrifugation parameters allowed the production of large MOF-layers with controlled thickness (Fig. 2). The flakes were adsorbed by dip-coating at room temperature on Si/SiO_2_ (300 nm) (ESI† for experimental details). Fig. 2 shows AFM *vs.* optical images of MOF-layers with thicknesses ranging from 2 to 30 nm. XPS characterization of these flakes deposited on highly oriented pyrolytic graphite (HOPG) agrees with the data previously published by us^29^ confirming their composition. The control over the thickness of the MOF layers is achieved by adjusting the sonication time in the exfoliation procedure (ESI† for experimental details). The statistical analysis of the thickness of the MOF-layers shows the excellent control of the exfoliation within the sonication time and the production of homogeneous materials (Fig. S1†).

Free-standing flakes of [Cu(μ-pym_2_S_2_)(μ-Cl)]_*n*_ are obtained by dip-coating with a water suspension of the polymer at 55 °C on a Si/SiO_2_ (300 nm) substrate with predefined circular wells (diameters ranging from 0.5 to 3 μm and 400 nm depth; Fig. S2 in ESI†). The increase of temperature of the suspension helps to reduce the surface tension of water minimizing the formation of the meniscus in the wells and makes the solution slightly more volatile improving the ratio of free-standing *vs.* collapsed MOF layers.

**Fig. 3:**
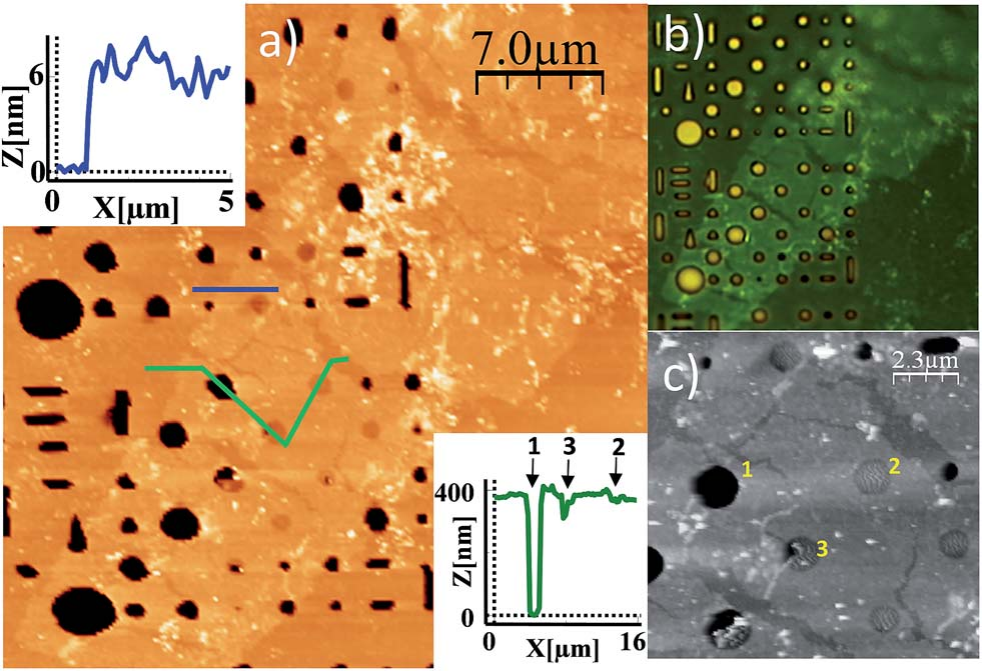
Morphological characterization of the free-standing layers of [Cu(μ-pym_2_S_2_)(μ-Cl)]_*n*_·*n*MeOH obtained on Si/SiO_2_ (300 nm) substrates with wells. (a) AFM topographic image of a large suspended flake of [Cu(μ-pym_2_S_2_)(μ-Cl)]_*n*_·*n*MeOH in an array of circular wells (400 nm depth). The topography displays the layer covering several circular wells, and the profiles taken along the flake standing on SiO_2_ (blue line) and over the holes (green line), showing the areas where the sheet is suspended (2 and 3) and not suspended (1). Note how in this later case the layer completely collapses. The percentage of covered holes is about 46%. (b) Optical image of the same area than in (a). (c) Zoom area of the AFM image showing hole uncovered (1), fully covered (2) and partially covered (3) by flake of MOF [Cu(μ-pym_2_S_2_)(μ-Cl)]_*n*_·*n*MeOH.

The adjustment of withdrawal speed and solution concentration seems to be critical to obtain free-standing flakes during dip-coating. Inspection by optical microscopy revealed polymer flakes covering several holes of the substrate (Fig. 3b). Importantly, the existence of suspended layers implies that we are not facing the deposition of random material, or some kind of precipitation process, we are really delaminating the crystals.

Atomic force microscopy (AFM) images^30^ (Fig. 3a and c) confirm this observation and provide a narrow height distribution for the flakes that ranges between 4–8 nm corresponding, to 5 to 10 layers of [Cu(μ-pym_2_S_2_)(μ-Cl)]_*n*_·*n*MeOH, with lateral dimensions *ca.* 300–3600 μm^2^. Although in the ideal case such sheets consist of single monolayers, they are often manifested as incompletely exfoliated flakes comprising a small number (<10) of stacked monolayers.

Additionally the AFM images reveal holes where the flake is perfectly suspended and others where it has collapsed, probably as a consequence of the capillary forces introduced by the solvent during evaporation. The high ratio of holes with suspended layers indicates a strong tendency of this large polymer flakes to withstand these forces. As a matter of fact, if a water suspension of graphene oxide flakes is deposited by drop-casting on a holed surface, they also exhibit an even higher tendency to collapse as a consequence of the capillary forces.^31^ These results show these flakes are mechanically stable to be spanned over the holes and give qualitative insights into the robustness of the sheets that further will be tested by their mechanical characterization.

### Mechanical properties of free-standing flakes

Mechanical stability is a relevant topic for 2D materials. The mechanical properties are sensitive to defects and thus they can be used as an indicator for the structural integrity and stability of the layers towards their application potential and device fabrication.^35^

**Fig. 4:**
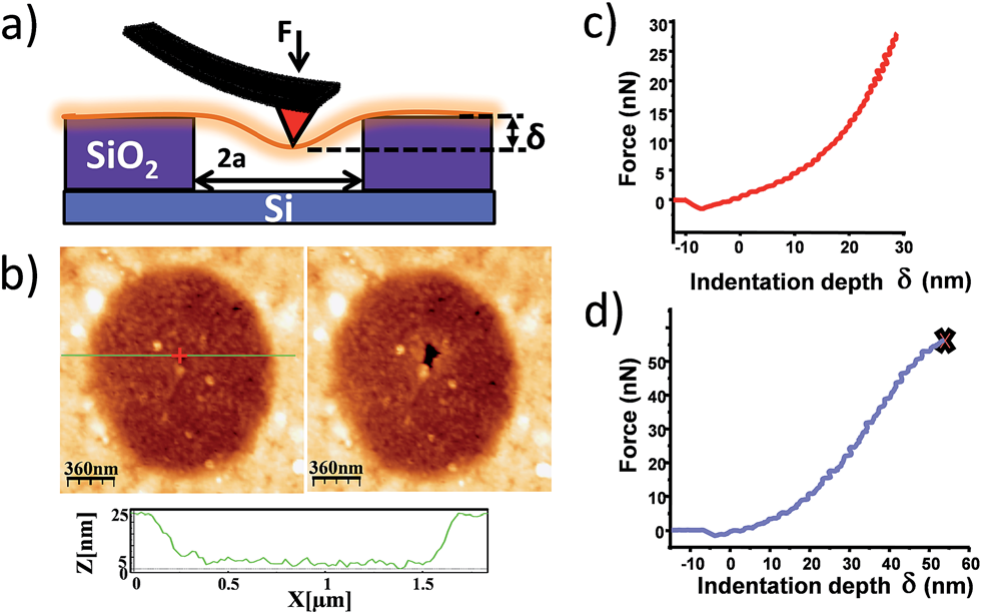
(a) Schematic representation of nanoindentation experiment on suspended MOF membrane. (b) Dynamic mode AFM image of a covered hole (1 μm in diameter) by [Cu(μ-pym_2_S_2_)(μ-Cl)]_*n*_·*n*MeOH membrane and a fractured membrane from indentation. The graph corresponds to the topographic profile along the green line in the image. (c) Elastic response test results on a suspended MOF membrane displaying the loading/unloading force curve of elastic stiffness. (d) Fracture test results on a suspended MOF membrane showing the breaking force curve (fracture load is indicated by X mark).

Mechanical characterization of the suspended flakes was performed using a standard nanoindentation set up with AFM.^32^ Fig. 4a displays a scheme of the experimental set up. The mechanical load applied by the AFM tip can be considered to a good approximation as a punctual force F that produces an indentation *δ* given by:1
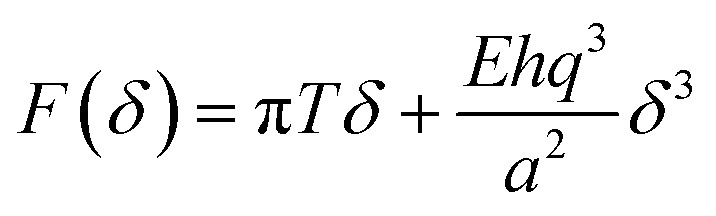
Where *F* is the loading force, *δ* is the indentation at the central point, *T* is the pretension accumulated in the sheet during the preparation procedure, *q* ≅ 1, *h* is the layer thickness and *a* is the drumhead radius. *E* is the Young's modulus, a fundamental parameter that characterizes the stiffness of a material. Fitting eqn (1) to curves measured in up to 7 different drumheads in 8 layer flakes yielded values of *E* and *T* of 5 ± 0.5 GPa and 0.12 ± 0.09 N m^–1^, respectively. Eqn (1) is a good approximation since the tip radius (≈25 nm) is much smaller than the hole radius (≈1000 nm) used to suspend the flakes in the experiments.

The same experimental set up can also be used to estimate the breaking force *F*_0_ and breaking strength given by σ* = [*F*_0_ *E*/(*h*π*R*_tip_)]^1/2^ where *R*_tip_ is the tip radius (we have used two different AFM tips with radii 25 and 15 nm).^32^ We measured breaking forces of about 40 nN yielding σ* = 1 ± 0.4 GPa. Notice that the expression for the breaking force is for a linear material that tends to overestimate this figure.

Density functional calculations were carried out on [Cu(μ-pym_2_S_2_)(μ-Cl)]_*n*_·*n*MeOH (ESI† for details) yielding *E* ranging from 3.4 GPa for the polymer single layer without solvent molecules, up to 4.1 GPa when methanol molecules are considered.^36^ Both figures are in good agreement with the experimental results.

Table 1 summarizes the results. The Young's modulus measured for the MOF studied here is the lowest reported so far, being approximately 200 times lower than the one measured for pristine graphene. The breaking strength follows a similar tendency being 150 times lower than the one for graphene, yet it was still possible to suspend very thin layers of this compound from solution, where the capillary forces tend to collapse the membranes. Therefore, contrary to what one might expect, the weak strength bonds are enough to retain the 2D-layer structure as mechanically coherent entities.

**Table 1:** Young modulus of different 2D materials

Material	Value (GPa)	Ref.
Graphene	800–1000	32, 33
MoS_2_	350–450	9
Hexagonal boron nitride	250	8
Graphene oxide	200	6, 7
Carbon nanosheets	10–50	34
2D clays	20	10
[Cu(μ-pym_2_S_2_)(μ-Cl)]_*n*_	5	This work

### Luminescence studies

Studies of additional physical properties are of the greatest interest and never reported before for layers of MOFs of nanometer thickness. Therefore, since optical properties of single crystals of [Cu(μ-pym_2_S_2_)(μ-Cl)]_*n*_·*n*MeOH were previously reported by us,^29^ we decided to study how these optical properties persist to isolated layers. To this end, samples of [Cu(μ-pym_2_S_2_)(μ-Cl)]_*n*_·*n*MeOH were imaged in reflection to locate large ‘flakes' and suitable candidates for spectroscopy were identified by their apparent colour in the optical microscope, which is observed due to interference effects in very thin few layers flakes on the Si/SiO_2_ substrate. These flakes were subsequently characterized by AFM in order to determine their thickness and thereby to calculate the number of layers in each flake – the individual flakes are easily recognizable by their individual shapes and orientations relative to each other. After locating a flake, confocal spectral images were obtained by rastering the laser focus across the selected area of the sample.

**Fig. 5:**
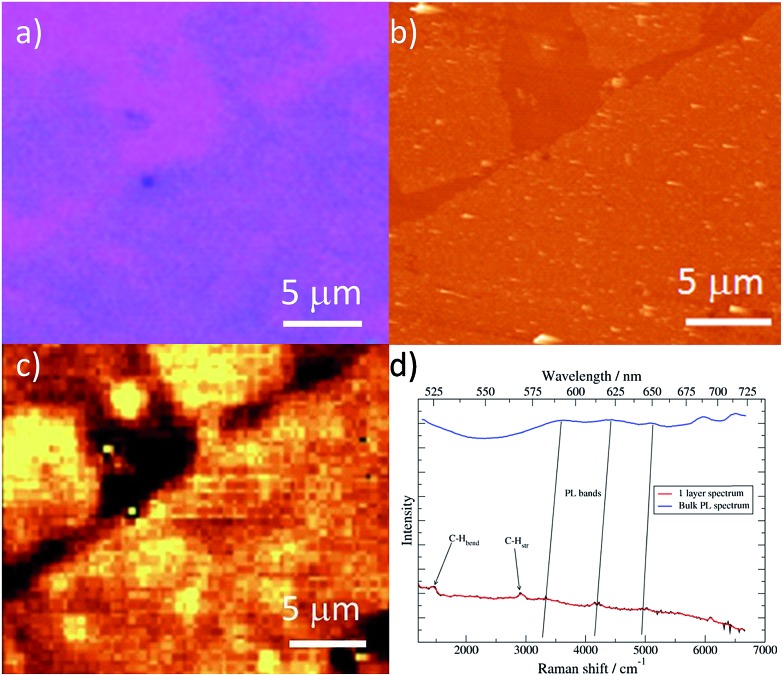
Optical characterization of a large single flake of [Cu(μ-pym_2_S_2_)(μ-Cl)]_*n*_·*n*MeOH. (a) and (b) Optical image and AFM topographic image of a large flake of [Cu(μ-pym_2_S_2_)(μ-Cl)]_*n*_·*n*MeOH on SiO_2_, respectively. (c) Confocal image of flakes of [Cu(μ-pym2S2)(μ-Cl)]_*n*_·*n*MeOH on an Si/SiO_2_ substrate. The colour-scale reflects the intensity within a Raman shift of 100 cm^–1^ from the elastically scattered light, *i.e.*, the image represents reflected light. Selected flakes, identified in the AFM image in panel b. (d) Raman/luminescence spectra of [Cu(μ-pym_2_S_2_)(μ-Cl)]_*n*_·*n*MeOH as a bulk sample on an Si/SiO_2_ substrate and as ‘flakes' comprising single layers on the Si/SiO_2_ substrate. The *x*-axis is shown as both the Raman shift with respect to the incident light (*λ*_ex_ = 488 nm) and as absolute wavelength because different features are due to Raman bands (shift with the laser wavelength) and PL (independent of laser wavelength). The individual spectra have been scaled and offset in order to display all the spectra on plot. The layer spectra were obtained using a confocal microscope (Witec CRM200); the bulk spectrum was recorded on a 48000s (T-Optics) spectrofluorometer from SLM-Aminco (*λ*_ex_ = 531 nm).

Each image corresponds to 10^4^ individual spectra and the colour-scale is determined by integration of the spectra over a certain range of Raman shift. Fig. 5c shows an image of elastically-scattered light (<100 cm^–1^ Raman shift from the laser line 488 nm). Several large flakes are clearly visible and the identical flakes can also be identified in the optical and AFM images of Fig. 5a and b, respectively. Height analysis of the AFM images revealed that these flakes correspond to a single layer (height *ca.* 2 nm).^29^

Fig. 5d shows the Raman/luminescence spectra of the sample from Fig. 5a–c. It is clear that there are similarities as well as differences between the flake spectra and the bulk spectrum. Three bands (indicated by grey vertical lines) are present in the bulk and flake spectra near 580 nm, 615 nm and 650 nm. We assign these features to PL because the same emission wavelength was observed with a different excitation wavelength of 531 nm (in ESI Fig. S9† feature (iii)); they are blue-shifted in the flake spectra compared to the bulk spectrum by about 5–10 nm. In Fig. 5d we also observe bands centred at 526 nm (1470 cm^–1^ Raman shift) and 569 nm (2920 cm^–1^ Raman shift). Similar features appear in Fig. S9 (ESI†), although more details are visible because the longer wavelength excites PL to a lesser extent. Because these bands appear at fixed energy with respect to the laser, we assign them to Raman processes described by C–H bending and C–H stretching modes of the 2D-MOF and associated solvent (MeOH) molecules.

Density functional calculations (B3LYP/6-31G(d)) (ESI† for details) of the vibration modes of the ligand confirm that there are groups of vibrations near 1470 cm^–1^ (Fig. S9 in ESI†) which are associated with normal modes that are combinations of sp^2^ C–H bending and either C–C or C–N modes of the ring. These features are sharp in the bulk spectrum, but are broad and weak for flakes, which can be understood in terms of the partial loss of translational symmetry in the thin layers. In addition, it should be noted that solvent exchange effects MeOH/H_2_O are known for this compound^29^ and these may have a strong influence on the Raman features in this region because solvent molecules present in the bulk crystal are less easily exchanged than those present near the surface, which will exchange even in air. These solvent effects could explain the differences in behaviour of [Cu(μ-pym_2_S_2_)(μ-Cl)]_*n*_ compared to graphite^37^ and MoS_2_ (ref. 38) with respect to the evolution of Raman spectra with sample thickness.

Other properties appear less sensitive to solvent effects. The luminescence properties of flakes on silicon oxide with thickness ranging from single, few to many layers, shift to the red, but otherwise do not significantly change as the number of layers increases (see also Fig. S8 and S9†).

**Fig. 6:**
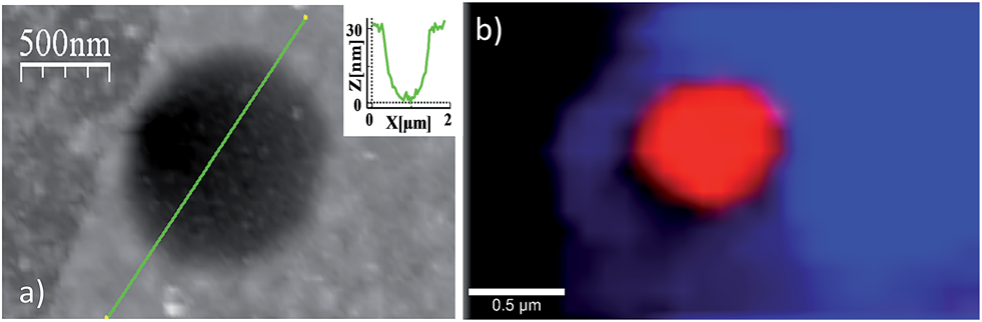
(a) AFM image showing a free-standing [Cu(μ-pym_2_S_2_)(μ-Cl)]_*n*_·MeOH layer adhered to the vertical hole for 30 nm in depth as displays the topographic profile along the well. (b) Photoluminescence image of the suspended layer showed in (a), where the luminescence of the free-standing layer (in red), the layer directly standing on SiO_2_ (in blue), and the SiO_2_ surface (in black) can be appreciated individually.

Finally, we also measured Raman spectra of flakes suspended over circular holes (Fig. 6 and S11†). It is observed that the spectrum of free-standing flakes are qualitatively the same, though with a slightly larger intensity, to those lying on the Si/SiO_2_ substrates previously analysed and do not arise from flake–substrate interactions.

## Experimental section

### General methods

All chemicals were of reagent grade and were used as commercially obtained. The reactions were carried out under dry argon atmosphere using Schlenk techniques and vacuum-line systems. The dipyrimidinedisulfide (pym_2_S_2_) ligand was prepared according to the published procedure.^39^ [Cu(μ-pym_2_S_2_)(μ-Cl)]_*n*_·*n*MeOH was prepared with a slight modification of the procedure already published.^29^

### Luminescence/Raman measurements

The spectral images were recorded on α300RA and CRM200 confocal Raman microscopes (Witec GmbH, Ulm, Germany). The 532 and 488 nm lines, respectively, of an Nd-YAG and an argon ion laser provided the excitation light and the emitted and/or scattered light passed through a Raman edge filter to remove elastically scattered light. The filtered light was collected by a multimode optical fibre that also served as the confocal pinhole. The objective was a (100×) lens with a numeric aperture of 0.95 and a 50 μm single mode fibre was used to supply the excitation light. Luminescence excitation and emission spectra of the solid [Cu(μ-pym_2_S_2_)(μ-Cl)]_*n*_·*n*MeOH were performed at 25 °C on a 48000s (T-Optics) spectrofluorometer from SLM-Aminco.

### AFM measurements

Atomic Force Microscope (AFM) techniques were used in dynamic mode using a Nanotec Electronica system operating at room temperature in ambient conditions. The images were processed using WSxM.^30^ For AFM measurements, commercial Olympus Si/N and Ti/Pt cantilevers were used with a nominal force constant of 0.75 N m^–1^ and 2 N m^–1^, respectively (ESI† for details). Mechanical characterization of the free-standing flakes was performed by indenting an AFM tip at the centre of the suspended area. Only membranes showing a flat and homogeneous surface were selected for the measurements. Curves acquired on the Si/SiO_2_ (300 nm) non-deforming substrate were used as a reference for calculating the applied force and the resulting deflection of the layers (indentation *δ*).

## Conclusions

In summary, we report the use of liquid phase exfoliation and dip-coating as a simple an efficient top-down method for the production of flakes of a metal–organic framework with lateral dimensions of hundreds of square microns and with an excellent control of the thickness. The isolated layers on SiO_2_ have been characterized by AFM and Raman, they show red emission, and this observation remains for isolated free-standing flakes. The mechanical characterization confirms the stability of these layers. To the best of our knowledge, this is first time that the physical properties of layers of a free-standing MOF are reported. Mechanical stability of layers based on MOFs^15^ will be a requirement for device fabrication. Herein we have shown a proof-of-concept on the feasibility of materials based on MOF-layers. Obviously, polymers such as covalent organic fragment-works (COFs) will be a source of 2D-materials in the near future.
