# Supplementary material for: Mechanical and optical properties of ultralarge flakes of a metal–organic framework with molecular thickness
Source: Chem Sci. 2015 Feb 16;6(4):2553–8. doi: 10.1039/c4sc03115f (PMC5647856; doi:10.1039/c4sc03115f)
Supplement: Supplementary file 1 [file SC-006-C4SC03115F-s001.pdf]

Electronic Supplementary Material (ESI) for Chemical Science.
This journal is © The Royal Society of Chemistry 2015

Electronic Supplementary Information

Mechanical and optical properties of ultralarge flakes of a metal-organic framework with molecular thickness

Cristina Hermosa, Benjamin R. Horrocks, José I. Martínez, Fabiola Liscio, Julio Gómez-Herrero,* and Félix Zamora*

Experimental Section

Materials and Methods

All chemicals were of reagent grade and were used as commercially obtained. The dipyrimidinedisulfide (pym_2S_2) ligand was prepared according to the published procedure.[1] The reactions were carried out under a dry argon atmosphere using Schlenk techniques and vacuum-line systems.

X-ray powder diffraction measurements. 2D-GIXRD images were recorded at the XRD1 beamline at the Elettra synchrotron facility at Trieste (Italy) using a monochromatic beam with a wavelength of 1 Å. The incident angle of the X-ray beam, α_i =0.1°, has been chosen close to the critical angle for the total reflection of the organic. The diffraction patterns have been recorded on a 2D camera (Pilatus detector) placed normal to the incident beam direction. X-ray beam, 0.2×0.2 (H×V) mm^2, probes a sample area of 5×0.2 mm^2 (footprint). Several images have been recorded translating the sample 0.5 mm in a direction perpendicular to the beam to have information on the sample homogeneity.

Luminescence/Raman Measurements. The spectral images were recorded on α300RA and CRM200 confocal Raman microscopes (Witec GmbH, Ulm, Germany). The 532 and 488 nm lines, respectively, of an Nd-YAG and an argon ion laser provided the excitation light and the emitted and/or scattered light passed through a Raman edge filter to remove elastically scattered light. The filtered light was collected by a multimode optical fibre that also served as the confocal pinhole. The objective was a (100 X) Lens with a numeric aperture of 0.95 and a 50 µm single mode fibre was used to supply the excitation light. The lateral spatial resolution of the α300RA instrument is close to the diffraction limit, that is, about 300 nm, while for the CRM200 instrument it is about 250 nm. For the α300RA instrument, the collected light was analysed by a spectrograph with typical settings of 600 lines mm^{-1} (grating), an integration time of 2 seconds per pixel, and 50 × 50 pixels for each image in the case of flakes and single spectrum with integration time of 2 seconds and 10 accumulations. The laser power was about 5 mW for the flakes and about 0.5 mW in the case of crystals. For the CRM200 instrument, the collected light was analyzed by a spectrograph with typical settings of 150 lines mm^{-1} (grating), an integration time of 0.5 s per pixel, and 100 × 100 pixels for each image. Individual spectra were obtained by co-adding and averaging across the image using the manufacturer-supplied software.

Luminescence excitation and emission spectra of the solid $[Cu(\mu\text{-}pym_2S_2)(\mu\text{-}Cl)]_n\cdot nMeOH$ were performed at 25 °C on a 48000s (T-Optics) spectrofluorometer from SLM-Aminco. A front face sample holder was used for data collection and oriented at 60° in order to minimize light scattering from the excitation beam on the cooled R-928 photomultiplier tube. Appropriate filters were used to eliminate Rayleigh and Raman scatters from the emission. Excitation and emission spectra were corrected for the wavelength dependence of the 450 W xenon arc excitation, but not for the wavelength dependence of the detection system. Spectroscopic properties were measured by reflection (front face mode) on finely ground samples that were placed in quartz cells of 1 mm path-length. No attempt was made to remove adsorbed or dissolved molecular oxygen from the materials. Reference samples that do not contain any fluorescent dopant were used to check the background and optical properties of the samples.

Synthetic Procedure. $[Cu(\mu\text{-}pym_2S_2)(\mu\text{-}Cl)]_n\cdot nMeOH$ was synthesized upon slow crystallization under argon flow at 20 °C of a solution formed from a mixture of pym_2S_2 0.062 g (0.28 mmol) in 8 mL of 1:1 MeOH:MeCN and of 0.048 g (0.28 mmol) of $CuCl_2\cdot 2H_2O$ in 6 mL of methanol. Upon one month, orange crystals of $[Cu(\mu\text{-}pym_2S_2)(\mu\text{-}Cl)]_n\cdot nMeOH$ were obtained, then filtered and washed with methanol and diethylether, and dried under vacuum (0.054 g, 41 % yield). Anal. Calcd. (%) for $C_9H_{10}ClCuN_4S_2O$: C, 30.57; H, 2.83; N, 15.85; S, 18.11; Found C, 29.97; H, 2.69; N, 16.06; S, 18.01 %. IR (KBr, cm^{-1}): 3403 (w), 3054 (w), 1550 (vs), 1373 (vs), 1253 (m), 1173 (s), 808 (m), 749 (s), 650 (m). The crystals obtained have typical dimensions about 125-60 x 45-80 x 35-70 μm^3, one order of magnitude higher than those obtained by faster evaporation synthesis. X-ray powder analysis confirms the structure of the crystals[2].

AFM Measurements. Atomic Force Microscope (AFM) techniques were use in dynamic mode using a Nanotec Electronica system operating at room temperature in ambient air conditions. The images were processed using WSxM (freely downloadable scanning probe microscopy software from www.nanotec.es).For AFM measurements, commercial Olympus Si/N and Ti/Pt cantilevers were used with a nominal force constant of 0.75 N/m and 2 N/m, respectively.
The surfaces used for AFM were, SiO_2 300 nm (IMS Company) and Si/SiO_2 (300 nm thickness) substrates with predefined wells with diameters ranging from 0.5 to 3 μm and 400 nm in depth, use for indentation experiments.
Surface preparations. In order to obtain reproducible results, very flat substrates were used with precisely controlled chemical functionalities, freshly prepared just before the chemical deposition. SiO_2 surfaces were sonicated for 15 min. in acetone and 15 min. in 2-propanol and then dried under an Argon flow. The Si / SiO_2 (300 nm) substrates with wells used for indentation experiments were previously treated with oxygen plasma to enhance the flakes adhesion on the substrate.
AFM Sample preparation. 0.5 mg of $[Cu(\mu\text{-}pym_2S_2)(\mu\text{-}Cl)]_n\cdot nMeOH$ was dispersed in 1 mL of H_2O milli-Q and sonicated with an ultrasonication bath (Elma, 37 kHz, 380 W) at different times, 60, 75 and 90 min, in order to modulate the thickness of the flakes. Thereafter, the resulting suspension was ultra-centrifuged (MPW-350R centrifuge) at 9000 rpm for 5 min. at 10 °C. The resulting solution was diluted with milli-Q water to a concentrations ranging between 10^{-1} to 10^{-4} mg/mL. The diluted solutions were adsorbed on SiO_2 substrates by dip-coating deposition for 10 min. at 20 °C and then dried under an Argon flow. In the case of SiO_2 (300 nm)/Si substrates with wells dip-coating warm deposition (55 °C) was carried out. Dip-coating at 55 °C helps reducing the surface tension of water preventing formation of meniscus in the wells and makes the solution slightly more volatile improving the ratio of free-standing MOF membranes. The adjustment of withdrawal speed and the solution concentration seems to be critical parameters to obtain free-standing flakes during dip-coating. Finally, to identify the exfoliated layers, the substrates were checked using optical microscopy and then characterized by AFM.

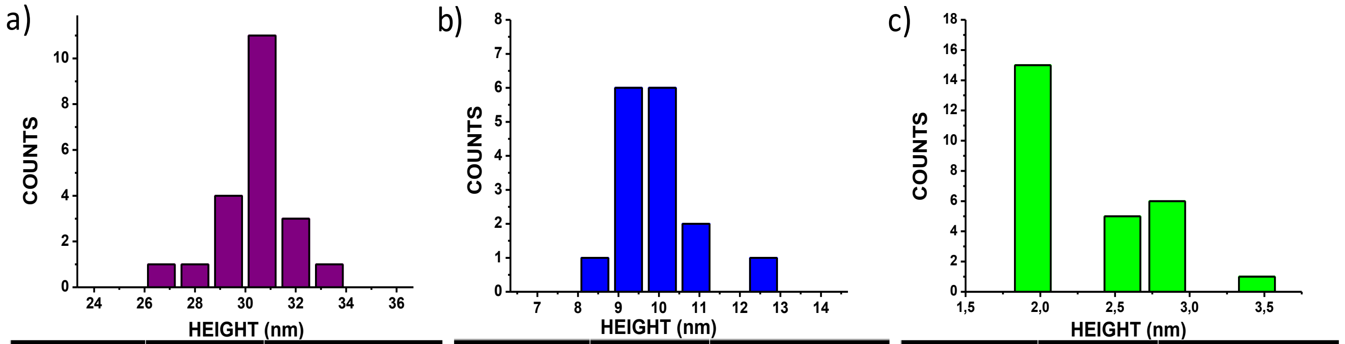

a)	Mean	Standard Deviation
Height (nm)	30,31429	1,36209

b)	Mean	Standard Deviation
Height (nm)	9,90937	0,94591

c)	Mean	Standard Deviation
Height (nm)	2,37037	0,47216

Figure S1. Analysis of the distribution in the thickness of the 2D flakes produced at different ultrasonic time: 60 min. (a), 75 min. (b), and 90 min. (c).

AFM indentation experiments. The substrates were fabricated from 6 inch silicon wafers (As-doped, resistivity 1 – 3.5 mΩ·cm). A 300 nm thick silicon oxide was grown by dry oxidation. The back of the wafer was coated with a 0.8 μm thick layer of AlSi(1%)Cu(0.5%). The silicon oxide was patterned using projection optical lithography and reactive ion etching (RIE). A chip was designed with patterns of different shapes and with critical dimensions of 0.5 μm, 1 μm, 1.5 μm and 3 μm, and separations between patterns of 2.5 μm and 3.5 μm (Figure S2). The design pattern was repeated many times in the chip, together with additional navigation marks, in order to easily locate the position of the individual flakes drumheads by optical microscopy and by AFM. The dimensions of the chip are 4 mm x 4 mm.

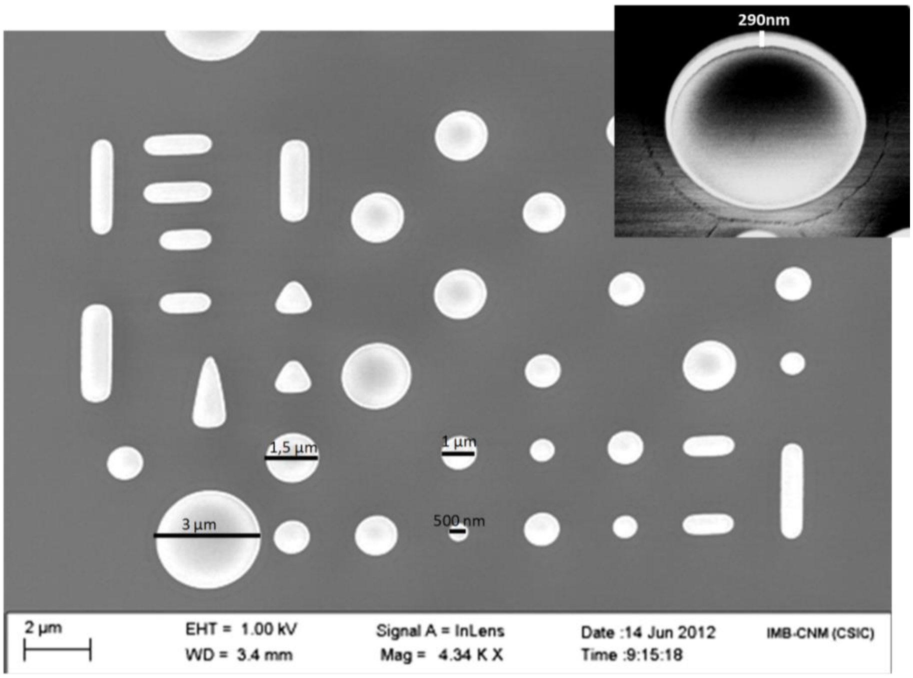

Figure S2. SEM image of one part of the chip after all the processes and a zoom in one of the holes to appreciate the verticality of the walls.

Suspended flakes on Si/SiO_2 (300 nm) substrates with circular wells were localized by an optical microscopy and then characterized by AFM imaging in dynamic mode in order to avoid damage to the samples. The topographic images determine the thickness of the flakes and therefore the number of layers in each flake. AFM height profile of the $[Cu(\mu\text{-}pym_2S_2)(\mu\text{-}Cl)]_n\cdot nMeOH$ flakes taken along the wells confirm the presence of a free-standing structure (Figure3a/4b).

Mechanical characterization of the free-standing flakes. It was performed by indenting an AFM tip at the centre of the suspended area. Only membranes showing a flat and homogeneous surface (without bubbles or wrinkles) were selected for the measurements. Curves acquired on the Si/SiO_2 (300 nm) non-deforming substrate were used as a reference for calculating the applied force and the resulting deflection of the layers (indentation δ).

In order to determinate E_{2D}, repeated loading/unloading curves at low indentations (low forces) were carried out on different membranes. The curves on the same membrane were observed overlap, indicating fully elastic deformations under these conditions and completely reversible behaviour. The slope of the $F(\delta)$ curves does not change after repeated deformations, proves the absence of permanent damage.

Two different methods were used to calculate the breaking force or maximum stress at the central point:

a) Once the data for elastic properties of the membrane were recorded, the flakes were once again indented, but this time to failure.

b) Only one indentation at high forces was carried out on the membrane.

Data sets from these two different protocols yielded similar values.

AFM topographies were imaging subsequently to each indentation to test the condition of the analysed membrane.

Force vs. indentation curves. The force-displacement data were processed to determine the elastic properties of the $[Cu(\mu\text{-}pym_2S_2)(\mu\text{-}Cl)]_n\cdot nMeOH$ membranes. To calculate the deformation of the flakes, the contribution due to the cantilever and sample displacement must be subtracted. When the tip and sample are in contact, the elastic deformation of the flake (δ), the defection of the AFM cantilever (Δz_c) and the sample displacement (Z) is related by:

$$\delta = Z - \Delta z_c$$

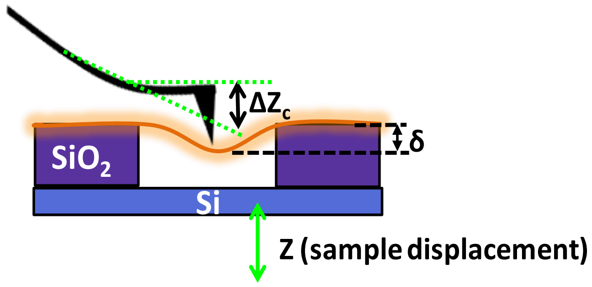

The force applied is related to the cantilever deflection as: F = Kcantilever * Δz_c
Therefore, the indentation on the flakes is calculated from the differences of the relative displacement of the samples and the tip on the non-deforming substrate, used as a reference.
The F (δ) curves provide access to the properties of the membranes (Figure S3).

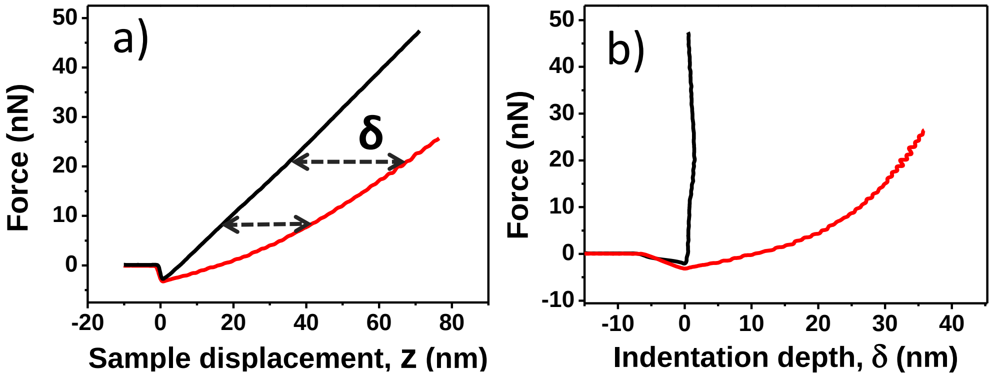

Figure S3. a) Force vs. sample displacement curve acquired on the Si/SiO_2 (300nm) substrate (black) and in the centre of the suspended flake (red) b) Force *vs.* indentation curve obtained. In Figure S2 it can be appreciated that in the rigid substrate the indentation is zero. Colour code is the same for both curves.

Two different cantilevers were used for the mechanical testing, with different tip radii and covered material as reported in the table. The corresponding cantilever spring constants were calibrated for each individual cantilever following Sader's method[3]. Similar breaking strengths were measured with both cantilever types.

Cantilever type	Nominal tip radius (nm)	Tip material	Stiffness (N/m)
Olympus OMCL-TR400PSA	25	Silicon nitride	0.7
Olympus OMCL-AC240TM	15	Si covered withTi/Pt	2

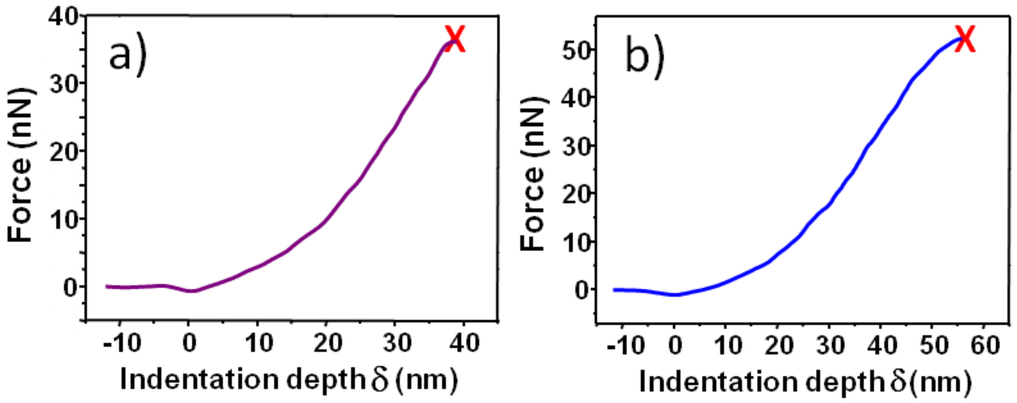

Figure S4. Fracture curves acquired in the centre of the suspended flake using **a)** Ti/Pt tip K= 2 N/m (Rectangular cantilever with tetrahedral probe) and, **b)** Si/N rip K= 0.7 N/m (Rectangular cantilever with pyramidal probe). The fracture load is indicated at the position marked “X”.

2. Results

X-ray powder diffraction. Figure S5 shows the diffraction peaks of the [Cu(μ-pym$_2$S$_2$)(μ-Cl)]$_n$·nMeOH before and after sonication treatment (90 min.) with the experimental conditions previously described for AFM preparation. No structural changes are detected confirming the integrity of the material after the sonication process.

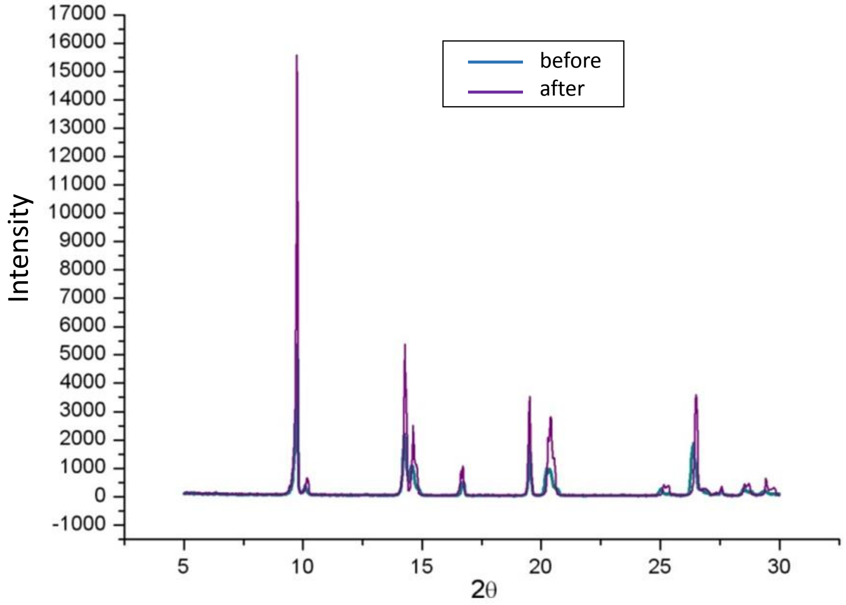

Figure S5. X-ray powder diffraction of [Cu(μ-pym$_2$S$_2$)(μ-Cl)]$_n$·nMeOH before (blue line) and after (purple line) sonication (ultrasonication Elma bath 37 kHz, 380 W) in H_2O (90 min.) treatment.

2DGIXRD analysis. Figure S6 shows the *2D* Grazing Incidence X-ray Diffraction (GIXRD) detector image collected on the same substrate sample used for the mechanical properties characterization.. A Bragg peak coming from the [Cu(μ-pym$_2$S$_2$)(μ-Cl)]$_n$·nMeOH layers structure appears at q= 6.4 nm^{-1}; it corresponds to the interlayer distance of (110) planes (d_{110}= 0.94 nm) where Cu atoms are located (Figure S7). The ring shape, characteristic of polycrystalline sample, ascribes from the folding and corrugation of several [Cu(μ-pym$_2$S$_2$)(μ-Cl)]$_n$·nMeOH layers, which make the diffraction from crystalline grain differently orientated allowed.

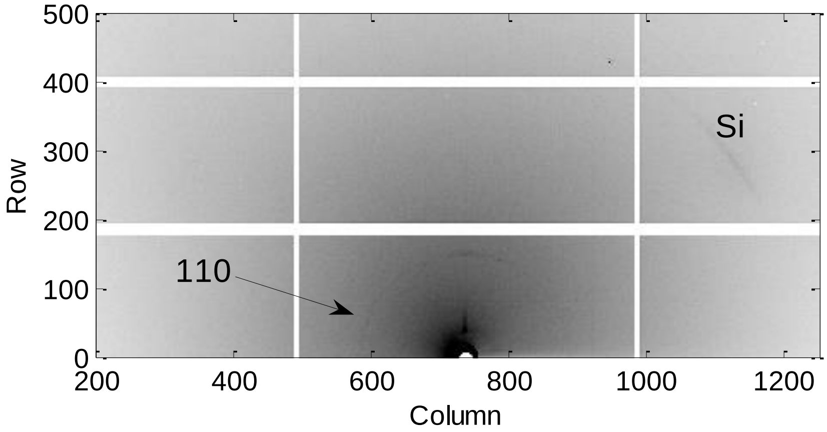

Figure S6. 2D Pilatus area detector images showing GIXRD signal coming from layers of [Cu(μ-pym$_2$S$_2$)(μ-Cl)]$_n$·nMeOH deposited on SiO$_2$/Si surface.

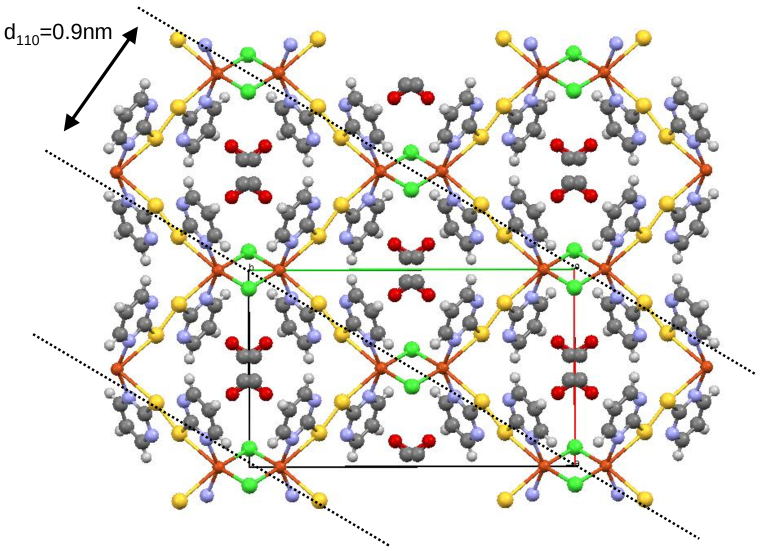

Figure S7. Schematic view {110} planes of a layer of [Cu(μ-pym$_2$S$_2$)(μ-Cl)]$_n$·nMeOH structure.

Raman-PL Luminescence experiments. The samples of [Cu(μ-pym$_2$S$_2$)(μ-Cl)]$_n$·nMeOH were imaged in reflection to locate large 'flakes' and suitable candidates for spectroscopy were identified by their apparent colour in the optical microscope, which is observed due to interference effects in very thin samples of a few layers on the Si/SiO$_2$ substrate. These flakes were subsequently characterized by AFM in order to determine their thickness and thereby to calculate the number of layers in each flake – the individual flakes are easily recognizable by their individual shapes and orientations relative to each other -. After locating a flake, confocal spectral images were obtained by rastering the laser focus across the selected area of the sample. Each image corresponds to 10^4 individual spectra and the colour-scale is determined by integration of the spectra over a certain range of Raman shift as given in the text or below.

(a) (b)
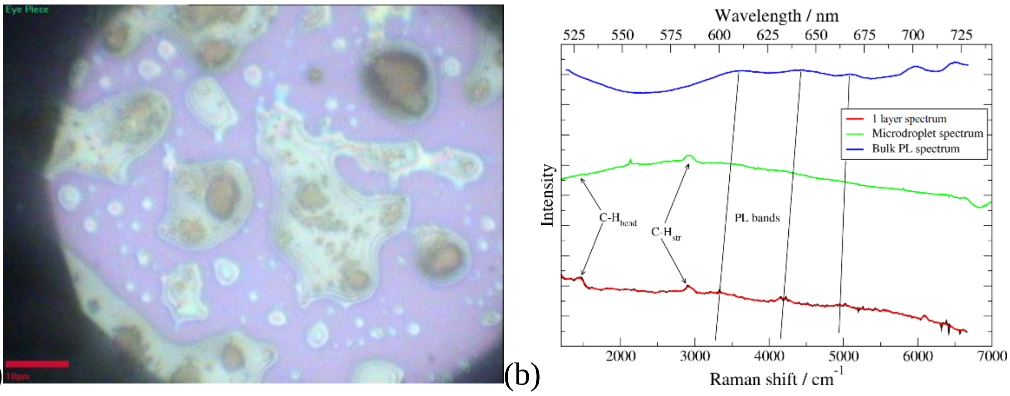

Figure S8. (a) Optical micrograph of the "microdroplets" on an Si/SiO$_2$ substrate. These appear to comprise flakes dried as a droplet rather than lying flat on the substrate. The scale bar is 10 μm. (b) Raman-luminescence spectra from Figure 5d of the main text, but including the microdroplet spectrum (green line).

Figure 5d (main text) shows the Raman/luminescence spectra of the sample from Figure 5a,b. As well as flakes, we also identified small 'microdroplets' of 2D-MOF on the Si/SiO$_2$ substrate (Figure S8). These were clearly much thicker (typically a few microns based on observing their (non-confocal) optical image and adjusting the focus of the objective) than the flakes and had the appearance of dried droplets with a high content of flakes; they showed more intense Raman/luminescence spectra and we consider them to represent an intermediate situation between the bulk solid and the flakes. The spectra of the flakes were obtained by averaging the spectra from the pixels on Figure 5c. It is clear that there are similarities as well as differences between the flake & droplet spectra on the one hand and the bulk spectrum on the other. In particular the bulk spectra show PL features near 680 nm and 720 nm which are not present in the other spectra, although it should be noted that the bulk sample was studied with a different excitation wavelength of 531 nm. However there are three bands (indicated by grey vertical lines on Figure 5d) which are present in the bulk, microdroplet and flake spectra near 580 nm, 615 nm and 650 nm. These are blue-shifted in the microdroplet and flake spectra compared to the bulk spectrum by about 5-10 nm. The blue-shift is larger for the thinner flake sample; this can be understood on the basis of quantum confinement if these features are PL transitions. Some of these bands can also be observed with 531 nm excitation in Figure S9 (indicated as feature (iii)). The observation of the same emission wavelength at a different excitation wavelength suggests these features are due to vibronic transitions associated with the PL.

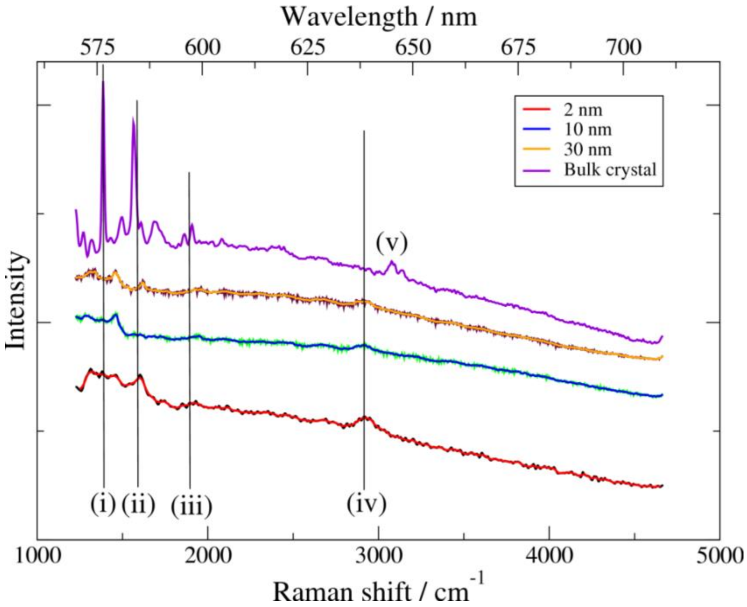

Figure S9. Raman/luminescence spectra of the 2D MOF showing the variation of the spectra with the thickness of the sample. The samples were 2, 10, 30 nm height on Si/SiO_2 substrates and a bulk crystal. The x-axis is shown as both the Raman shift with respect to the incident light (λ_{ex} = 531 nm) as wavelength because different features are due to Raman bands (shift with the laser wavelength) and PL (independent of laser wavelength). The individual spectra have been scaled and offset in order to display all the spectra on plot. Each spectrum is shown as raw data overlaid with an 11-point moving average smoothed curve. The data below about 1000 cm^{-1} are dominated by features due to the Si substrate and are therefore omitted. The spectra were obtained using a confocal microscope (Witec α300RA) and the layer thicknesses were determined by atomic force microscopy.

In Figure 5d we also observe bands centred at 526 nm (1470 cm^{-1} Raman shift) and 569 nm (2920 cm^{-1} Raman shift). Similar features appear in Figure S9 ((i),(iv)), although more details are visible because the longer wavelength excites less PL and a higher resolution grating was used. Because these bands appear at fixed energy with respect to the laser, we assign them to Raman processes described by C-H bending and C-H stretching modes of the 2D-MOF.

Density functional calculations (B3LYP/6-31G(d) (see below) of the vibration modes of the ligand show that there are two groups of vibrations (labelled (i) and (ii) in Figure S9) which are associated with normal modes that are combinations of sp^2C-H bending and either C-C or C-N modes of the ring. These features are sharp in the bulk spectrum, but are broad and weak for all the layer samples 2, 10 & 30 nm (Figure S9). This can be understood in terms of the partial loss of translational symmetry in the thin layers. Feature (iv) in Figure S9 and the corresponding band in Figure 5d are sp^3C-H stretching Raman modes. Such modes are not present in the ligand, but indicate the presence of organic molecules, possibly methanol, which remain bound to the layers. It is worth noting that band (ii) of Figure S9 overlaps with a similar feature in the Raman spectrum of liquid methanol.[4] The bands due to the sp^2C-H stretches of the ligand were only observed in the bulk crystal (v), but a consideration of their intensity relative to the C-H bends indicates they are too weak to observe in the layers.

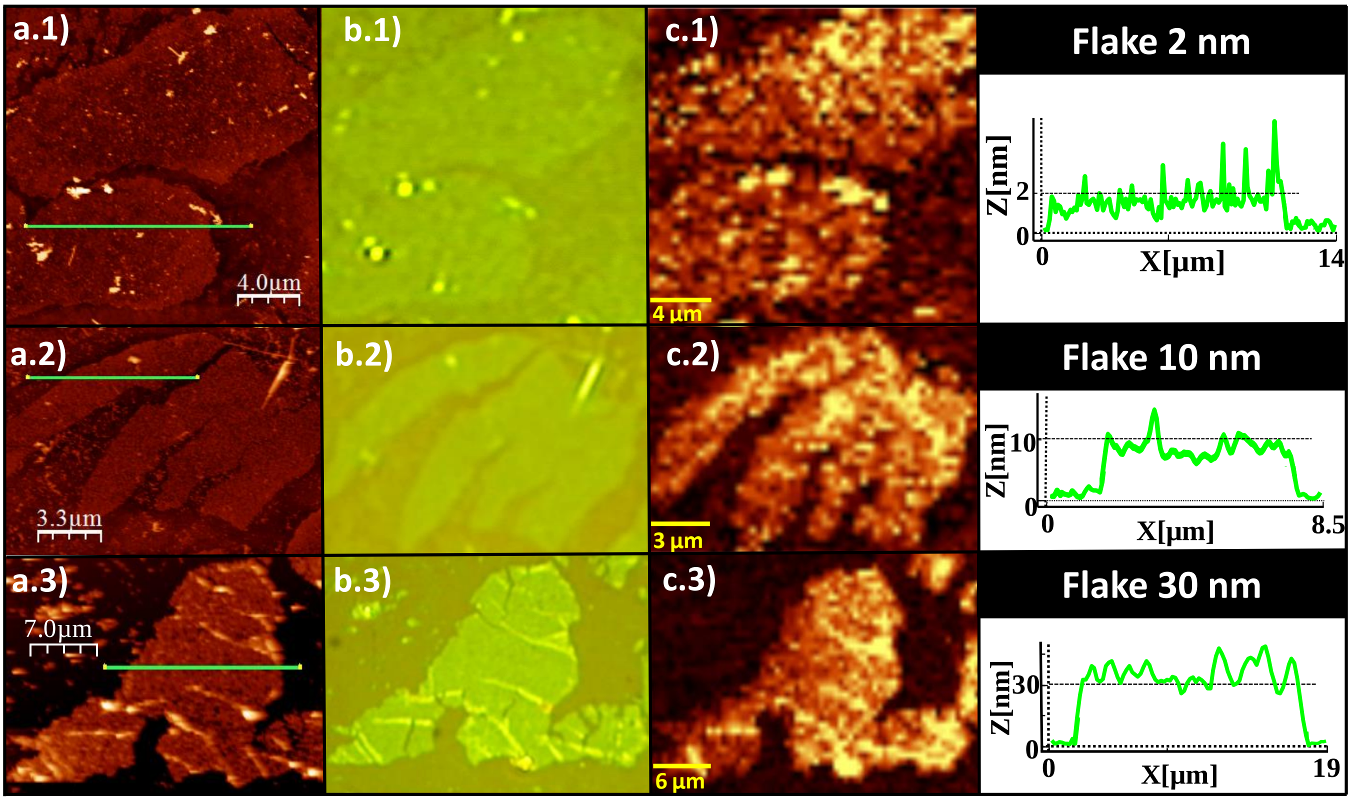

Figure S10. (a1-a3) AFM topographic images and their height profiles (showing thickness ranging from: single layer (2 nm, top), 14 layers (10 nm, middle) and 48 layers (30 nm, bottom), **(b1-b3)** the corresponding optical images, and **(c1-c3)** Raman/luminescence spectral images of the 2D-MOF showing the variation of the spectra of samples with different thickness on Si/SiO_2 substrates. The individual spectra have been scaled and offset in order to display all the spectra on plot. Each spectrum is shown as raw data overlaid with an 11-point moving average smoothed curve. The data below 1000 cm^{-1} are dominated by features due to the Si substrate and are therefore omitted. The spectra were obtained using a confocal microscope (Witec α300RA) and the layer thicknesses were determined by atomic force microscopy.

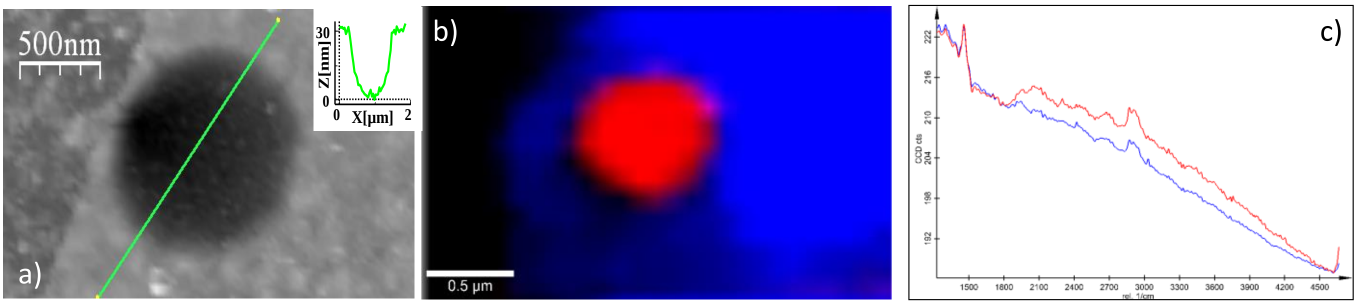

Figure S11. a) AFM image showing a free-standing layer adhered to the vertical hole for 30 nm in depth as displays the topographic profile along the well. **b)** Photoluminescence image of the suspended layer showed in **a**), where it can appreciate its luminescence in the hole (red) and in the non-suspended area (blue). Image made by mapping resulted in a spectrum shown in **c). c)** Raman spectra of the layer adsorbed on the non-deforming substrate (blue line) and in the hole with the free-standing flake (red line). The colour code is according with the image.

Figure S11 presents further examples of Raman/PL spectral images and demonstrates that the spectra of free-standing flakes are qualitatively the same, though with a slightly larger intensity, as those lying on the Si/SiO_x substrate and discussed in detail above.

DFT Calculations of Raman spectra. Quantum chemical calculations of the Raman spectra of the ligand were carried out using the GAMESS(US) program.[5] The geometries were first optimized using B3LYP as implemented in GAMESS(US) and second order perturbation theory (MP2) both with 6-31G(d) basis sets. The 'seminumerical' option for calculating the hessian (using analytical gradients) was employed and

the method for calculation of the Raman intensities was that of reference [6]. The vibrational wavenumbers were scaled according to the values suggested in the NIST database.[4]

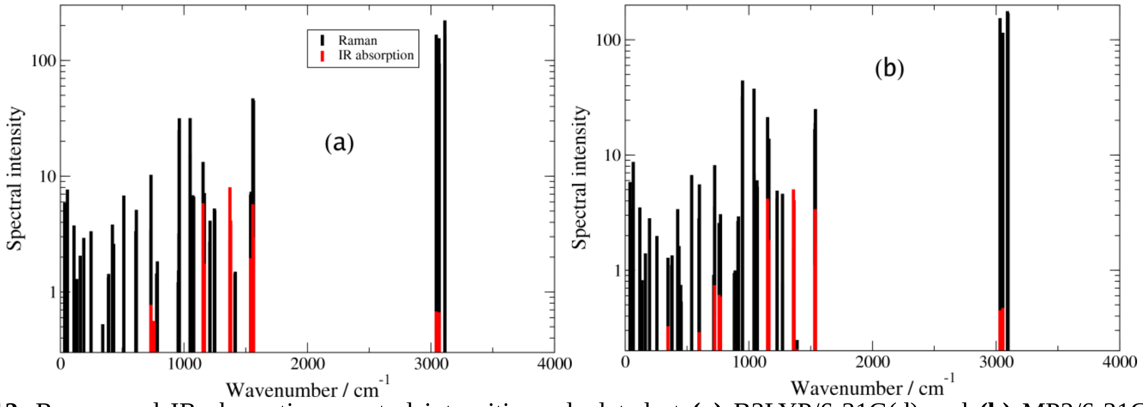

Figure S12. Raman and IR absorption spectral intensities calculated at **(a)** B3LYP/6-31G(d) and **(b)** MP2/6-31G(d) levels using GAMESS(US)[5]. Raman intensities are in units of Å^4 amu^{-1} and IR intensities are in units of D^2 amu^{-1} Å^{-2} and both are plotted on a logarithmic scale.

Theoretical Calculation of the Young Modulus E and Poisson ratio *ν*

In order to shed some light on the mechanical properties of the system from the theoretical point of view, we have followed a theoretical protocol reported by Cadelano and Colombo in reference [7] (and references therein) to calculate the Young modulus *E* and the Poisson ratio *ν* of the delaminated MOF $[Cu(\mu\text{-}pym_2S_2)(\mu\text{-}Cl)]_n$ and $[Cu(\mu\text{-}pym_2S_2)(\mu\text{-}Cl)]_n{\cdot}nMeOH$ single layers.

Method. Our multiscale approach benefits from continuum elasticity (used to define the deformation protocol[7] aimed at determining the elastic energy density of the investigated systems), and first-principles atomistic calculations (used to actually calculate such an energy density and the corresponding elastic moduli). Atomistic simulations have been performed by Density Functional Theory (DFT) as implemented in the QUANTUM ESPRESSO package.[8] The exchange-correlation effects have been accounted through the generalized gradient approximation (GGA) within the Perdew-Burke-Ernzerhof (PBE) parameterization.[9] Rabe Rappe Kaxiras Joannopoulos (RRKJ) ultrasoft pseudopotentials[10] have been used to model the ion-electron interaction in the H, C, N, O, S, Cl and Cu atoms. A plane-wave basis set with kinetic energy cutoff as high as 30 Ry was used, and the Brillouin zone (BZ) has been sampled by means of a [**2**×**4**×**1**] Monkhorst-Pack grid,[11] guaranteeing a full convergence in energy and density. The atomic positions of the investigated delaminated samples have been extracted from the geometries previously reported[2], and fully optimized by using damped dynamics and periodically-repeated simulation cells (with the following cell parameters: *a*=17.4 Å, and *b*=11.8 Å). Accordingly, in order to avoid any possible perpendicular inter-layer interaction, a vacuum spacing between perpendicular adjacent sheets in the supercell geometry greater than 16 Å was considered.

The Young modulus of the delaminated structures under consideration, MOF $[Cu(\mu\text{-}pym_2S_2)(\mu\text{-}Cl)]_n$ and $[Cu(\mu\text{-}pym_2S_2)(\mu\text{-}Cl)]_n{\cdot}nMeOH$ single layers, has been obtained from the *strain-vs-energy* curves, corresponding to suitable deformations applied to both samples (Figure S13). The corresponding simulation cell (dashed line in panels of Figure S13) contained 88 and 112 atoms for the clean (left panel) and methanol (right panel) forms, respectively, and they correspond to delaminated sheets with ideal bulk-crystal stoichiometry. As discussed in more detail in reference [7] (and references therein), for any deformation the magnitude of the strain is represented by a single parameter ζ. Thus, the *strain-vs-energy* curves have been carefully generated by varying the magnitude of ζ in steps of 0.001 up to a maximum strain ζ_{max} = ±0.02. The reliability of the above computational procedure has been previously proved by the estimated values for the Young modulus (*E*) and the Poisson ratio (*ν*) of other relevant 2D systems such as graphene (corresponding to 0% of hydrogen coverage), respectively 349 Nm^{-1} and 0.15, which are in excellent agreement with recent literature,[12] or C-graphane (corresponding to 100% of hydrogen coverage), respectively 219 Nm^{-1} and 0.21, agreeing with data already reported.[12a]

On the other hand, $[Cu(\mu\text{-}pym_2S_2)(\mu\text{-}Cl)]_n$ monolayers show an orthorhombic symmetry, which causes an anisotropic linear elastic behaviour. The two-dimensional elastic energy density (per unit of area) for

systems with orthorhombic symmetry can be expressed as[13]:

$$U = \frac{1}{2}\mathcal{C}_{11}\epsilon_{xx}^2 + \frac{1}{2}\mathcal{C}_{22}\epsilon_{yy}^2 + \mathcal{C}_{12}\epsilon_{xx}\epsilon_{yy} + 2\mathcal{C}_{44}\epsilon_{xy}^2, \quad (1)$$

where x and y indicate the two cartesian directions along the XY 2D monolayer lattice, and ϵ_{ij} ($i, j = x$ or y) is the infinitesimal strain tensor. Making use of the linear elastic constants $\mathcal{C}_{11}, \mathcal{C}_{22}, \mathcal{C}_{12}$ and $\mathcal{C}_{44}$ of Eq. (1) by simply imposing the isotropy condition ($\mathcal{C}_{11} = \mathcal{C}_{22}$), and the Cauchy relation ($2\mathcal{C}_{44} = \mathcal{C}_{11} - \mathcal{C}_{12}$) [13], the Young modulus E and the Poisson ratio ν can be straightforwardly evaluated as $E = \frac{(\mathcal{C}_{11}^2 - \mathcal{C}_{12}^2)}{\mathcal{C}_{11}}$, and $\nu = \frac{\mathcal{C}_{12}}{\mathcal{C}_{11}}$, respectively (see reference [7] and references therein). This means that E and ν can be directly obtained from the linear elastic constants $\mathcal{C}_{ij}$, in turn computed through *strain-vs-energy* curves corresponding to suitable homogeneous in-plane deformations. Only two in-plane deformations should be, in principle, applied in order to obtain all the independent elastic constants, namely: **i)** an uniaxial deformation along the ***x***; and **ii)** an hydrostatic planar deformation. Nevertheless, for the validation of the isotropicity condition, two more in-plane deformations must be further applied: **iii)** an axial deformation along the ***y*** direction; and **iv)** a shear deformation. The strain tensors corresponding to applied deformations depend on the unique scalar strain parameter ζ,[12a, 12b] so that the elastic energy of strained structures defined in Eq. (1) can be written as $U(\zeta) = U_0 + \frac{1}{2}U^{(2)}\zeta^2 + O(\zeta^3)$,where U_0 is the energy of the unstrained configuration. Since the expansion coefficient $U^{(2)}$ is related to the elastic constants $\mathcal{C}_{ij}$, a straightforward fit provides the full set of linear moduli for both delaminated systems.

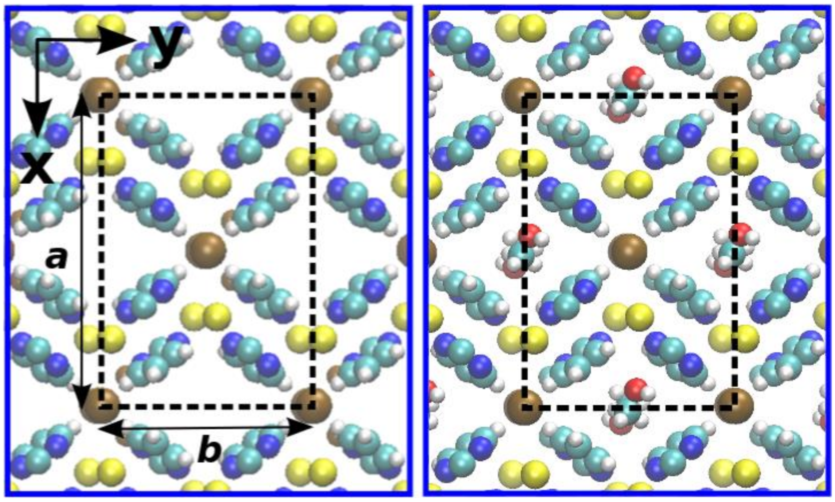

Figure S13. (Colour online) Pictorial top view representation of the delaminated $[Cu(\mu\text{-}pym_2S_2)(\mu\text{-}Cl)]_n$ (left panel) and $[Cu(\mu\text{-}pym_2S_2)(\mu\text{-}Cl)]_n\cdot nMeOH$ (right panel) single layers. Dashed lines represent the simulation cell (with **a**=17.4 Å, and **b**=11.8 Å). Hydrogen, carbon, nitrogen, oxygen, sulphur, chlorine and copper atoms are indicated by white, light blue, dark blue, red, yellow, dark brown and light brown spheres, respectively.

Results. The synopsis of the calculated elastic constants $\mathcal{C}_{ij}$, Young modulus E, and Poisson ratio ν, for both delaminated $[Cu(\mu\text{-}pym_2S_2)(\mu\text{-}Cl)]_n$ and $[Cu(\mu\text{-}pym_2S_2)(\mu\text{-}Cl)]_n\cdot nMeOH$ single layers here investigated is reported in Table S1.

Deviations from the isotropic elastic behaviour are quantitatively predicted by the calculation of the $\mathcal{A} = \frac{4\mathcal{C}_{44}}{(\mathcal{C}_{11} + \mathcal{C}_{22} - 2\mathcal{C}_{44})}$ ratio, which should be 1 for ideally isotropic systems. Data reported in Table S1 provide $\mathcal{A} =$ 1.01 (dimensionless) for both delaminated monolayered systems, thus confirming their elastic isotropicity.

Table S1. Independent elastic constants C_{ij} and Young modulus E (in GPa units), and Poisson ratio ν (dimensionless), are shown for both delaminated MOF [Cu(μ-pym$_2$S$_2$)(μ-Cl)]$_n$ and [Cu(μ-pym$_2$S$_2$)(μ-Cl)]$_n$·nMeOH single layers.

	**[Cu(μ-pym$_2$S$_2$)(μ-Cl)]$_n$**	**[Cu(μ-pym$_2$S$_2$)(μ-Cl)]$_n$·nMeOH**
C_{11}	23.2	28.5
C_{22}	23.2	28.5
C_{12}	8.1	8.3
C_{44}	7.6	9.6
E	3.4	4.1
ν	0.35	0.29

References

[1] R. Leino, J. E. Lonnqvist, *Tetrahedron Lett.* **2004**, *45*, 8489-8491.

[2] A. Gallego, C. Hermosa, O. Castillo, I. Berlanga, C. J. Gómez-García, E. Mateo-Marti, J. I. Martínez, F. Flores, C. Gómez-Navarro, J. Gómez-Herrero, S. Delgado, F. Zamora, *Adv. Matter.* **2013**, *25*, 2141–2146.

[3] J. E. Sader, J. W. M. Chon, P. Mulvaney, *Rev. Sci. Instrum.* **1999**, *70*, 3967-3969.

[4] http://cccbdb.nist.gov/vibscalejust.asp (accessed, 23rd May, 2013).

[5] a) M. W. Schmidt, K. K. Baldridge, J. A. Boatz, S. T. Elbert, M. S. Gordon, J. H. Jensen, S. Koseki, N. Matsunaga, K. A. Nguyen, S. J. Su, T. L. Windus, M. Dupuis, J. A. Montgomery, *J. Comput. Chem.* **1993**, *14*, 1347-1363; b) M. S. Gordon, M. W. Schmidt, (Eds.: C. E. Dykstra, G. Frenking, K. S. Kim, G. E. Scuseria), Elsevier, Amsterdam, **2005**, pp. 1167-1189.

[6] A. Komornicki, J. W. Mciver, *J. Chem. Phys.* **1979**, *70*, 2014-2016.

[7] E. Cadelano, L. Colombo, *Phys. Rev. B* **2012**, *85*, 245434.

[8] P. Giannozzi, S. Baroni, N. Bonini, M. Calandra, R. Car, C. Cavazzoni, D. Ceresoli, G. L. Chiarotti, M. Cococcioni, I. Dabo, A. Dal Corso, S. de Gironcoli, S. Fabris, G. Fratesi, R. Gebauer, U. Gerstmann, C. Gougoussis, A. Kokalj, M. Lazzeri, L. Martin-Samos, N. Marzari, F. Mauri, R. Mazzarello, S. Paolini, A. Pasquarello, L. Paulatto, C. Sbraccia, S. Scandolo, G. Sclauzero, A. P. Seitsonen, A. Smogunov, P. Umari, R. M. Wentzcovitch, *J Phys. Condens. Mat.* **2009**, *21*, 395502.

[9] J. P. Perdew, K. Burke, M. Ernzerhof, *Phys. Rev. Lett.* **1997**, *78*, 1396-1396.

[10] a) A. M. Rappe, K. M. Rabe, E. Kaxiras, J. D. Joannopoulos, *Phys. Rev. B* **1990**, *41*, 1227-1230; b) N. Mounet, N. Marzari, *Phys. Rev. B* **2005**, *71*, 205214.

[11] D. J. Chadi, M. L. Cohen, *Phys. Rev. B* **1973**, *8*, 5747-5753.

[12] a) E. Cadelano, P. L. Palla, S. Giordano, L. Colombo, *Phys. Rev. B* **2010**, *82*, 235414; b) E. Cadelano, P. L. Palla, S. Giordano, L. Colombo, *Phys Rev Lett* **2009**, *102*, 235502; c) G. Gui, J. Li, J. X. Zhong, *Phys. Rev. B* **2008**, *78*; d) K. N. Kudin, G. E. Scuseria, B. I. Yakobson, *Phys. Rev. B* **2001**, *64*, 235406; eF. Liu, P. M. Ming, J. Li, *Phys. Rev. B* **2007**, *76*, 064120.

[13] a) H. B. Huntington, *The Elastic Constants of Crystals*, Academic Press, New York, **1958**; b) L. D. Landau, E. M. Lifschitz, *Theory of Elasticity*, Butterworth Heinemann, Oxford, **1986**.
